# Use of a *Clostridioides difficile* Murine Immunization and Challenge Model to Evaluate Single and Combination Vaccine Adjuvants Consisting of Alum and NKT Cell-Activating Ligands

**DOI:** 10.3389/fimmu.2021.818734

**Published:** 2022-01-14

**Authors:** Gillian A. Lang, Kaylee Norman, Souwelimatou Amadou Amani, Tyler M. Shadid, Jimmy D. Ballard, Mark L. Lang

**Affiliations:** Department of Microbiology and Immunology, University of Oklahoma Health Sciences Center, Oklahoma City, OK, United States

**Keywords:** adjuvant, Alhydrogel™, α-galactosylceramide, CD1d, immunization, Alum

## Abstract

Adjuvant combinations may enhance or broaden the expression of immune responses to vaccine antigens. Information on whether established Alum type adjuvants can be combined with experimental CD1d ligand adjuvants is currently lacking. In this study, we used a murine *Clostridioides difficile* immunization and challenge model to evaluate Alum (Alhydrogel™), α-galactosylceramide (α-GC), and one of its analogs 7DW8-5 singly and in combination as vaccine adjuvants. We observed that the Alum/α-GC combination caused modest enhancement of vaccine antigen-specific IgG1 and IgG2b responses, and a broadening to include IgG2c that did not significantly impact overall protection. Similar observations were made using the Alum/7DW8-5 combination. Examination of the impact of adjuvants on NKT cells revealed expansion of invariant NKT (iNKT) cells with modest expansion of their iNKTfh subset and little effect on diverse NKT (dNKT) cells. Side effects of the adjuvants was determined and revealed transient hepatotoxicity when Alum/α-GC was used in combination but not singly. In summary these results showed that the Alum/α-GC or the Alum/7DW8-5 combination could exert distinct effects on the NKT cell compartment and on isotype switch to produce Th1-driven IgG subclasses in addition to Alum/Th2-driven subclasses. While Alum alone was efficacious in stimulating IgG-mediated protection, and α-GC offered no apparent additional benefit in the *C. difficile* challenge model, the work herein reveals immune response features that could be optimized and harnessed in other vaccine contexts.

## Introduction

The range of vaccine adjuvants currently deployed in the clinical setting is limited. Aluminum-based ‘Alum’ formulations are and have been for decades, those most commonly used. Commensurate with development of prototype adjuvants that operate through diverse mechanisms, there is interest in development of combination adjuvant platforms (1–3). Such adjuvant combinations may enhance or broaden the expression of immune responses to vaccine efficacy or bring other benefits such as dose sparing.

Alum has a positive track record for efficacy when a Th2-driven humoral immune response is desired, but it does not stimulate good Th1-driven humoral (IgG2c) or cellular (CD8^+^ CTL) immunity [reviewed in (4, 5)]. Alum-enhanced humoral responses require damage-associated molecular patterns (DAMPs) such as uric acid to be released from Alum-damaged cells (6). Uric acid in turn acts in an inflammasome-dependent manner to boost antigen presentation by APCs such as dendritic cells (DCs) and Th cell priming (7, 8). Alum also leads to recruitment of numerous cell types including monocytes, eosinophils, neutrophils, Natural Killer (NK) cells, and Natural Killer T (NKT) cells to immunization sites (9).

Murine invariant NKT (iNKT) cells express a semi-invariant T cell receptor (TCR) combining a Vα14-Jα18 rearrangement with Vβ chains 8.2, 7, or 2 in mice (10–12). Human iNKT cells express a semi-invariant Vα24,Jα18,Vβ11 TCR (13). The TCR on murine and human iNKT cells recognizes the cell surface CD1d loaded with glycolipid ligands such as α-galactosylceramide (α-GC) on APCs (14). CD1d/glycolipid and TCR interactions facilitate activation of NKT cells leading to regulation of anti-microbial and tumor immunity, autoimmunity and self-tolerance (15). In studies by our group and others, it has been observed that immunization with iNKT-activating ligands enhance humoral and cellular immune responses to co-administered antigens (Ags), pathogens, or tumors (16–28). The α-GC glycolipid enhances adaptive immune responses to malaria, cancer, influenza, and bacteria (and their toxins) (15, 19, 20, 26–36). Although α-GC and its derivatives can induce a strong Th1 response *in vivo* (32, 34), in the context of humoral immunity, a more balanced Th1/Th2 response is also observed (16, 18, 19, 36). The α-GC adjuvant has a shorter track record than Alum, but is well tolerated in human subjects, and has shown some promise in boosting tumor-specific Cytotoxic T Lymphocyte (CTL) responses (37–39). Following intraperitoneal (i.p.) or intravenous (i.v.) delivery using a polysorbate vehicle, α-GC can over-stimulate iNKT cells and cause functional anergy (40, 41). However, subcutaneous delivery in the absence of polysorbate can avoid iNKT anergy (17). The 7DW8-5 molecule, an analog of α-GC also has potent adjuvant effects on adaptive immune responses (42, 43). 7DW8-5 has a higher affinity for CD1d than α-GC and consequently when delivered by the intramuscular route (i.m.) is retained locally unlike α-GC which disperses and exerts systemic effects (44).

Follicular helper iNKT (iNKTfh) cells represent a subset of iNKT cells that arise through proliferation and activation of a Bcl6 transcription factor-driven differentiation program (45–47). The iNKTfh subset is characterized as TCRβ^+^, CD1d^tetramer+,^CD44^hi^, CXCR5^hi^, PD-1^hi^ and secrete IL-4 and IL-21 (47, 48). Although a role in B cell help is evident for B cell responses, their precise contribution to T-dependent versus T-independent and primary versus recall/memory remains unclear (31, 46, 49).

Also related to iNKT cells, a group of CD1d-restricted diverse NKT (dNKT) cells has been described which are non-reactive with α-GC and express a variable TCR (50). The dNKT subset is protective in autoimmune diabetes, experimental autoimmune encephalomyelitis and Concanavalin A-induced hepatitis (51–53) and can suppress tumor immuno-surveillance (52). In contrast to iNKT cells that are known to mediate enhanced antibody (Ab) responses against foreign Ag, little is known about dNKT contributions to humoral immunity other than one report by our group documenting reduced responsiveness to Imject™ Alum adjuvant in mice lacking dNKT cells (54).

*C. difficile* is a Gram positive bacterium that is the leading cause of hospital derived infections, antibiotic-associated diarrhea and pseudomembranous colitis. *C. difficile* can also cause systemic disease that includes cardiotoxicity and multiple organ dysfunction (55, 56). One of the defense mechanisms and correlates of protection against *C. difficile* infection (CDI) includes a serum IgG response against *C. difficile* toxins A and B (TcdA and TcdB) (57–59). We have previously shown that both Alum (Imject™ and Alhydrogel™ (AL)) and the α-GC adjuvant can boost TcdB-specific Ab responses (36). IgG-dependent protection against a toxin challenge and a live pathogen challenge in a mouse infection model has been demonstrated following immunization against TcdB when Alhydrogel™ was used as an adjuvant (36, 60). Evidence is also accumulating that targeting of oligo- or polysaccharide antigens on *C. difficile* is a viable vaccine strategy (61, 62) and recent studies from our group showed that α-GC could enhance protection following vaccination with *C. difficile* polysaccharides (63). Although a combination toxin- and carbohydrate-based vaccine may be necessary for success in the clinic, toxin-based approaches allow a clear examination of the contribution of the humoral immune response to protection in challenge models.

In this study we evaluated Alum and α-GC adjuvants singly and in combination for their ability to enhance immunity and protection against *C. difficile* as compared to either adjuvant alone. We measured TcdB-specific humoral immunity, systemic toxicity, iNKT and dNKT expansion and differentiation into iNKTfh cells, as well as protection against a live *C. difficile* challenge. We show that a combination of α-GC and Alum can broaden the humoral immune responses and that 7DW8-5 can differentially stimulate iNKT and the Alum / {alpha}-GC combination iNKTfh expansion. However, there was little additional benefit with regard to protection against *C. difficile* disease and the Alum / {alpha}-GC combination was associated with transient hepatotoxicity. In summary the Alum/α-GC combination may be useful for broadening humoral immunity to some pathogens but may have limited utility for application to *C. difficile* toxin-based vaccines.

## Materials And Methods

### Ethics

This study was carried out in accordance with the recommendations in the Guide for the Care and Use of Laboratory Animals of the National Institutes of Health. All animal procedures were approved by the OUHSC Institutional Animal Care and Use Committee.

### Reagents

Key reagents were purchased as follows: HRP-conjugated anti-mouse IgM, IgG1, IgG2b, IgG2c, and IgG3 (Southern Biotech, Birmingham, AL); Biotin-conjugated anti-CXCR5 (2G8), and FITC-conjugated anti-CD4 (GK1.5) mAbs and APC-conjugated streptavidin were from BD Biosciences (San Jose, CA). The PE-Cy7-conjugated anti-PD-1 (RPMI-30), PE-conjugated anti-IgD (11-26c.2a), and BV421-conjugated anti-CD44 (IM7) mAbs were purchased from Biolegend (San Diego, CA). FcR-blocking mAb 2.4G2 was from BioXCell (Lebanon, NH). Alhydrogel™ (*In vivo*gen, San Diego, CA); α-GC (Axorra, Farmingdale, NY); 7DW8-5 (Diagnocine, Hackensack, NJ); 2,2’-azino-bis(3-ethylbenzothiazoline-6-sulphonic acid), ABTS (KPL, Gaithersburg, MD); Cefoperazone (Sigma, St. Louis, MO). *C. difficile* was cultured and TcdB and CTD purified as previously described [28]. The CTD-encoding region of *tcdb* gene (YP_001087135.1: nucleotides 4961–7111) from *C. difficile* strain VPI-10463 was codon optimized and cloned into pET15b (Genscript). The CTD gene was amplified using primers 5′-GATCATATGCTGTATGTGGGTAACCG-3′ and 5′-AACGGATCCTTATTCGCTAATAACCA-3′ containing *Bam*HI and *Nde*1 sites for cloning into pET15b. CTD (representing VPI-10463 TcdB_1651–2366_) was expressed in *Escherichia coli* BL21 star DE3 (Invitrogen) and purified by Ni^2+^ affinity chromatography (HisTrap, GE Life Sciences).

### Mice

Female C57Bl/6 mice were purchased from Charles River (Bethesda, MD, USA). Before experiments, all mice were housed under the same specific-pathogen free conditions. Mice were 6-8 week-old mice at the time of immunization and 10-12 weeks old at the time of antibiotic treatment and *C. difficile* infection.

### Adjuvant Dose and Immunizations

Previous work by our group documented that 1 to 4 µg α-GC when administered s.c. led to iNKT expansion and activation without over-stimulation (17), and with the higher dose boosting T-dependent humoral immune responses (16, 17). Similarly 100 µl of a 2% suspension of Alum (Alhydrogel™, *In vivo*gen, San Diego, CA) exerted a strong adjuvant effect on CTD-specific IgG responses in mice (36). Work by the Tsuji laboratory compared effects of 1 µg and 10 µg 7DW8-5 adjuvant on anti-hemagglutinin IgG responses and protection against influenza challenge in mice, showing good protection at the higher dose and more modest effects at the lower dose (42). For this study, Alum, α-GC, and 7DW8-5 doses were reduced to determine if suboptimal protection when adjuvants were administered singly could be enhanced by their combination. Mice were anesthetized with a vaporized 4% isoflurane/96% medical air mix and immunized subcutaneously (s.c). Unless indicated otherwise, mice received the following formulations: 10 µg of CTD in sterile phosphate-buffered saline (PBS), adsorbed to Alum (25 µl of the 2% suspension) (36); CTD mixed with 1 µg α-GC or 2 µg 7DW8-5 (49); CTD adsorbed to Alum and mixed with α-GC or 7DW8-5. For prime boost experiments, mice received NP-KLH adsorbed to Alum then NP-KLH mixed with 7DW8-5 14 days later (or vice versa). Where indicated, mice received adjuvants but not antigens, including in toxicity determinations. The timelines and vaccination schemes for this study are outlined in **Figure S1**).

### Flow Cytometry

Inguinal, and axillary lymph node cells and spleens were isolated by mechanical disruption and red blood cells were removed by hypotonic lysis with Tris-buffered Ammonium Chloride. The cells were suspended in RPMI media with 1% FBS. The cells were incubated with anti-FcR–blocking antibody (2.4G2, 20 µg/ml) for 5 min and stained with fluorochrome conjugated mAbs for 30 min at room temperature. Cells were then washed with ice-cold PBS three times (290 RCF, 5 min, 22°C) and fixed with 2% w/v paraformaldehyde in PBS. The cells were analyzed on a Stratedigm S1200Ex flow cytometer (Stratedigm, San Jose, CA). Data were analyzed with FlowJo software (Tree Star, Ashland, OR).

### Toxicity Assay

Mice were immunized s.c. with Alum, α-GC, 7DW8-5, Alum/α-GC, or Alum/7D5-8W in the amounts used for immunization and protection experiments. Heparinized blood samples were collected on days 1, 2 and 7. Blood samples were pooled (3 per group to obtain 150 µl for analysis) and tested within 1 hr using an Abaxa VetScan VS2 veterinary blood analyzer (Union City, CA) in conjunction with a Comprehensive Diagnostic test cartridge which measures the following: Albumin (ALB); Alkaline Phosphate (ALP); Alanine Transaminase (ALT); Amylase (AMY); Bilirubin (TBIL); Blood Urea Nitrogen (BUN); Calcium (CA); Phosphate (PHOS); Creatinine (CRE); Glucose (GLU); Sodium (NA+); Potassium (K+); Total Protein (TP); Globulin (GLOB).

### Bleeds

Blood samples were obtained by the retro-orbital route using heparinized capillary tubes. Erythrocytes were removed by centrifugation producing plasma samples that were stored at 4°C or -20°C as required.

### ELISA

To measure antigen-specific antibodies, ELISA Max™ enzyme-linked immunosorbent assay (ELISA) 96-well plates (Thermo Fisher Scientific, Rochester, NY). were coated with 10 µg/ml of antigen in Phosphate coating buffer (0.1 M Na_2_HPO_4_ in deionized water, pH=9.0) overnight at 4°C. Wells were blocked with 1% Bovine Serum Albumin in PBS-T (PBS 1X, 0.05% tween) for 2 hours at room temperature, and incubated overnight at 4°C with serially –diluted mouse sera. Wells were washed with PBS-T and then incubated for 1h with Horse-Radish Peroxidase (HRP)-conjugated IgM (1:5000), IgG1 (1:8,000), IgG2b (1:5000), IgG2c (1:5000), or IgG3 (1:5000). Wells were washed and developed for 5 min at room temperature with ABTS substrate (KPL, Gaithersburg, MD). A 10% w/v SDS solution was used to stop the reaction. Endpoint Ab titers were determined by measuring the O.D at 405 nm.

### Preparation of *C. difficile* Spores

In this study, the VPI 10463 strain of C*. difficile* was used for infection of mice (64). *C. difficile* VPI 10463 spores were prepared and isolated as previously described and all steps were performed anaerobically at 37°C. Briefly, pre-reduced Columbia Broth (BD) was inoculated with a single colony of bacteria. The culture was then transferred and grown in Clospore media, a liquid media that allow production of high titers of *C. difficile* spores (65). Spores were harvested and stored in sterile water.

### *C. difficile* Infection

*C. difficile* infection was induced in mice as previously described (60). Briefly, mice were treated with Cefoperazone sodium salt (Sigma-Aldrich, St. Louis, MO) at a final concentration of 0.5 mg/ml in distilled drinking water for five days followed by a two-day sterile water period. Mice were then orally gavaged with 10^5^ CFU of *C. difficile* VPI 10463 spores. The weights of the mice were monitored once per day for up to 14 days. Fecal samples were collected, and *C. difficile* bacteria were quantified on day 3 post gavage to assess bacterial burden.

### Fecal Bacteria Enumeration

Numbers of shed bacteria were quantified on day 3 post-gavage, unless otherwise indicated. Fecal pellets were homogenized with 1X PBS, serially diluted, plated on TCCFA and cultured under anaerobic conditions at 37°C. CFUs were counted within 24 and 48 hours (66).

### Statistics

Data were analyzed using GraphPad Prism 8.1 (La Jolla, CA, USA). A two tailed T-test or a Mann-Whitney test, and One-way ANOVA with Dunnett’s multiple comparison test were used for statistical analysis between two and multiple experimental groups respectively. A Two-way repeated measures ANOVA with Dunnett’s multiple comparisons test was used to determine statistical significance in weight loss over time.

## Results

### A Single Dose of Alum-Adsorbed CTD Affords Sufficient Protection Against *C. difficile* but Inclusion of CD1d Ligand Broadens the Humoral Immune Response to TcdB

**Figure 1:**
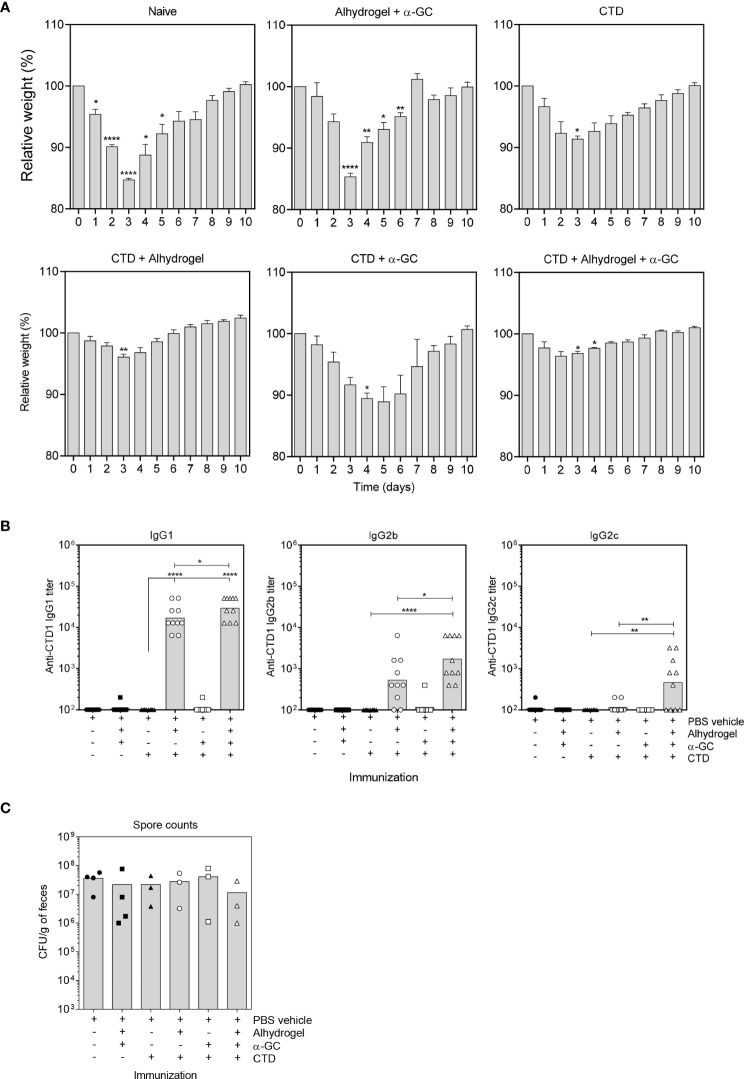
A single dose of Alum-adsorbed CTD affords sufficient protection against *C. difficile* but inclusion of α-GC broadens the humoral immune response to TcdB. **(A)** B6 mice were immunized s.c. as indicated with a single dose of each vaccine. After 28 days, mice were bled and treated with Cefoperazone for 5 days. After antibiotic withdrawal and provision of regular drinking water for 2 days, mice were orally gavaged with 5 x 10^4^ live *C. difficile* spores. Weights were then monitored daily. Graphs show mean ± SEM percentages of pre-infection weights for 5 mice per group. A Two-way repeated measures ANOVA with Dunnett’s multiple comparisons test was used to detect statistically significant differences in weight. Data are representative of two similar experiments. **(B)** Anti-CTD IgG1 (left), IgG2b (middle), and IgG2c (right) were detected by ELISA. Endpoint titers are shown, and each data point represents an individual mouse. Data from two pooled experiments is shown. Statistical significance was detected by ANOVA with Dunnett’s multiple comparison test. (*P < 0.05; **P < 0.01; ****P < 0.0001). **(C)** *C. difficile* spore counts in day 3 fecal pellets were determined as described in materials and methods. Pellets could not be collected from every mouse which was attributed to dehydration.

As expected, naïve mice exhibited severe weight loss over 3 days following *C. difficile* spore challenge before recovery over a further 7 day period (**Figure 1A**). Mice immunized with the Alum and α-GC adjuvants in the absence of antigen (CTD) had a similar disease course to naïve mice showing that the adjuvants afforded no protection in the absence of antigen. Immunization with the CTD antigen in the absence of adjuvant resulted in partial protection, blunting overall weight loss, but did not alter the course of disease. Immunization with CTD plus α-GC had a modest effect on weight loss and delayed maximum weight loss by 1-2 days. Immunization with the Alum-adsorbed CTD vaccine was strongly protective with minimal weight loss. Immunization with Alum-adsorbed CTD plus α-GC (referred to hereafter as the combination vaccine), had no further discernable effect on weight loss. The CTD-specific Ab response was dominated by IgG1 (**Figure 1B**). Alum-adsorbed CTD induced a strong IgG1 response whereas adjuvant alone, CTD alone, and CTD plus α-GC did not. The combination vaccine modestly increased IgG1 titers and the effects were statistically significant. Although the CTD-specific IgG2b responses were lower than that observed for IgG1, effects of the adjuvants singly and in combination were similar. In contrast, all vaccine modalities failed to induce an IgG2c response with the exception of the combination vaccine, which therefore broadened the humoral response. As expected, none of the immunization modalities affected bacterial burden (**Figure 1C**). This is because the vaccine targets secreted toxins which largely account for pathology associated with *C. difficile* infection. In a follow up experiment, CTD was tested in combination with a higher 4 µg α-GC dose and followed with a booster vaccine consisting of CTD only. In that experiment, our prior observations that α-GC could enhance the IgG response were observed (36, 49), confirming that α-GC was functional in our study (*data not shown*). These data demonstrate that inclusion of α-GC in a vaccine platform containing Alum has little effect on protection against *C. difficile* but does broaden the humoral immune response to include IgG2c.

**Figure 2:**
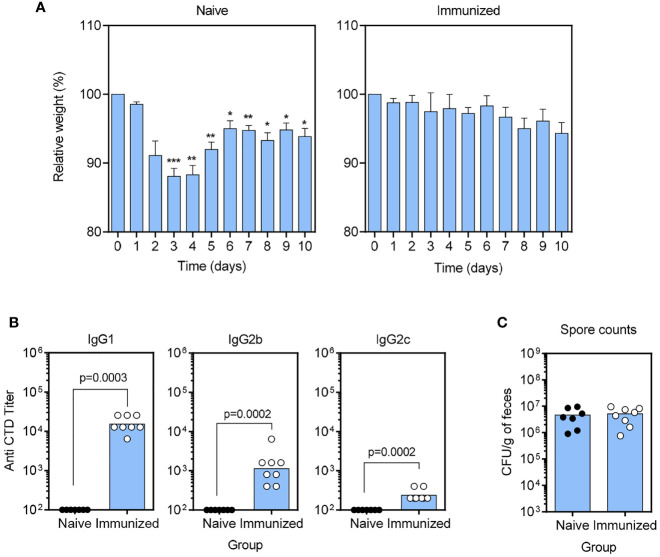
Inclusion of the α-GC analog 7DW8-5 in the CTD/Alum vaccine does not confer changes to IgG subclass or protection beyond that provided by α-GC. **(A)** B6 mice were immunized s.c. with PBS vehicle (naïve) a single dose of the CTD/Alum plus 7DW8-5 vaccine before antibiotic treatment and *C. difficile* spore challenge. Weights were then monitored daily. Graphs show mean ± SEM percentages of pre-infection weights (Naïve, n=7, Immunized, n=8). A Two-way repeated measures ANOVA with Dunnett’s multiple comparisons test was used to detect statistically significant differences in weight. (*P < 0.05; **P < 0.01; ***P < 0.001). **(B)** Anti-CTD IgG1 (left), IgG2b (middle), and IgG2c (right) were detected by ELISA. Endpoint titers are shown, and each data point represents an individual mouse. Statistical significance was detected by 2-tailed Mann-Whitney U test. **(C)** *C. difficile* spore counts in day 3 fecal pellets were determined as described in *Materials and Methods*.

We also tested a combination vaccine consisting of Alum-adsorbed CTD plus the functional α-GC analog 7DW8-5 to determine if there were discernable features from the response to the α-GC-containing vaccine (**Figure 2**). As expected, naïve mice lost a significant amount of body weight then mounted a partial recovery showing a more prolonged course of infection than in the previous experiment (**Figure 2A**). In contrast, immunized mice were protected from weight loss. The Ab titers were similar to that observed when using α-GC as the adjuvant with a high IgG1 titer, moderate IgG2b titer and low IgG2c titer (**Figure 2B**). *C. difficile* spore counts in the fecal pellets were unaffected by vaccination as expected (**Figure 2C**). These data show that 7DW8-5 did not result in changes to Ab profiles or protection beyond that induced by α-GC.

### NKT Cell Expansion With Single and Combination Adjuvants

**Figure 3:**
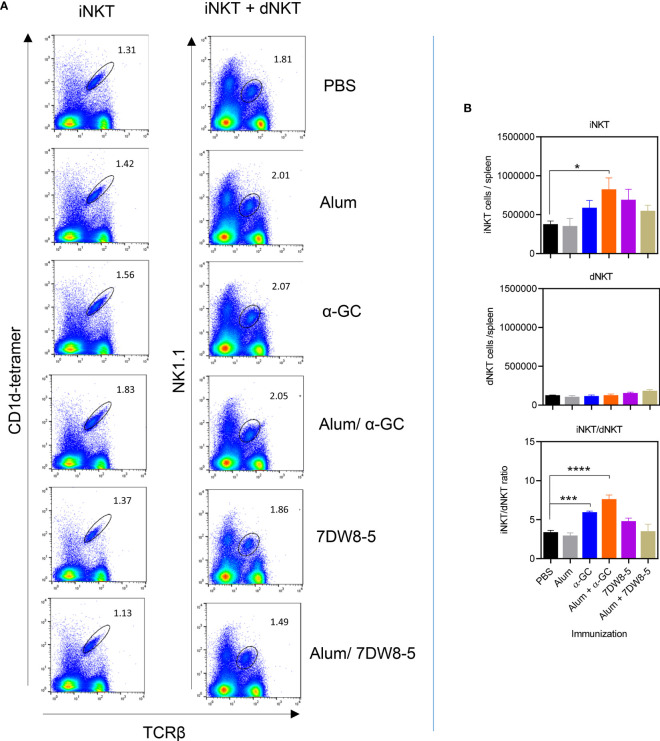
The Alum/α-GC combination adjuvant increases iNKT cell numbers and the iNKT/dNKT ratio. Mice were immunized as indicated and after 8 days, splenocytes prepared and examined by flow cytometry. **(A)** Shows representative flow cytometry plots of total NKT cells (iNKT and dNKT, left column) and of iNKT cells (right column). **(B)** Shows mean ± SEM absolute numbers of iNKT cells (upper panel) and dNKT cells (middle panel). dNKT numbers are calculated by subtracting the TcRβ^+^/tetramer^+^ population from the TCRβ^+^/NK1.1^+^ population. Also shown is the iNKT/dNKT ratio for absolute numbers (lower panel). Data show 3 pooled experiments with similar results (PBS, Alum n=12, α-GC, Alum/α-GC n=11, 7DW8-5, Alum/7DW8-5 n=3). One way ANOVA with Dunnett’s post-test was used to detect significant differences (*P < 0.05; ***P < 0.001; ****P < 0.0001).

Since α-GC and 7DW8-5 are well established to activate iNKT cells and humoral immune responses to Alum Imject adjuvant have a partial dependence on dNKT cells, we examined these populations by flow cytometry 8 days after adjuvant administration (**Figure 3** and **Figure S2**). Splenocytes were examined for TCRβ^+^, NK1.1^+^ lymphocytes which are consistent with iNKT and dNKT cells collectively. Using CD1d tetramers loaded with the PBS57 artificial α-GC ligand allows differentiation between the majority iNKT (TCRβ^+^, NK1.1^+^, tetramer^+^) and minority dNKT (TCRβ^+^, NK1.1^+^, tetramer^-^) populations. When Alum and α-GC were administered singly significant increases in the frequency of iNKT cells and dNKT cells were not observed. The Alum/α-GC combination led to an increase in iNKT cell frequency (**Figure 3A** and **Figure S3A**). Neither Alum and α-GC administered singly or combined led to significant increases in absolute numbers of iNKT cells and dNKT cells. However, α-GC and the Alum/α-GC combination led to a significant increase in the iNKT/dNKT cell ratio. 7DW8-5 alone or in combination with Alum had no significant effect on iNKT or dNKT numbers (**Figure 3B**).

**Figure 4:**
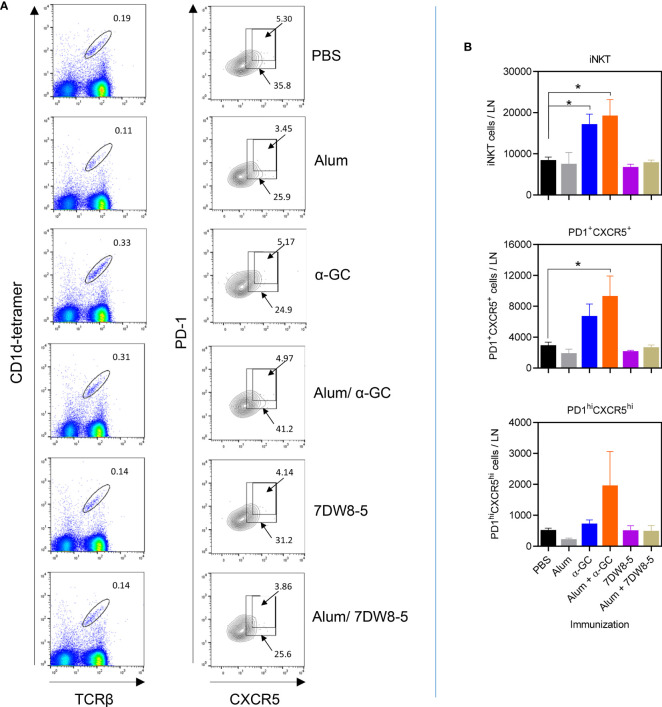
Expansion of iNKTfh cells following administration of single and combination adjuvants. Lymph nodes from mice described in this figure were analyzed by flow cytometry for iNKTfh cells. **(A)** Shows representative flow cytometry plots of CXCR5^+^/PD-1^+^ and CXCR5^hi^/PD-1^hi^ events after gating on TCRβ^+^/tetramer^+^ events. **(B)** Graphs show mean ± SEM absolute numbers of TCRβ^+^/tetramer^+^, CXCR5^+^/PD-1^+^ and CXCR5^hi^/PD-1^hi^ events. Data shown 2 pooled experiments with similar results (PBS, Alum n=6, α-GC, Alum/α-GC n=6, 7DW8-5, Alum/7DW8-5 n=3). One way ANOVA with Dunnett’s post-test was used to detect significant differences (*p < 0.05).

Lymph node cells were also analyzed for the presence of iNKTfh cells (**Figure 4**). The iNKTfh population can be defined as those expressing high levels of PD-1 and CXCR5 within the PD-1^+^/CXCR5^+^ population (47). Indeed we previously demonstrated that ablation of the Bcl6 transcription factor in the CD4^+^ lineage resulted in a selective loss of the PD-1^hi^/CXCR5^hi^ population (49). Alum did not cause detectable expansion of PD-1^+^/CXCR^+^ or PD-1^hi^/CXCR5^hi^ iNKT cells. The α-GC adjuvant and the Alum/α-GC combination did not cause statistically significant increases in iNKT frequencies or that of the PD-1^+^/CXCR5^+^ or PD-1^hi^/CXCR5^hi^ populations (**Figure 4A** and **Figure S3B**). However, α-GC and the Alum/α-GC combination caused statistically significant increases in the absolute numbers of iNKT cells, while Alum/α-GC significantly increased the PD-1^+^/CXCR5^+^ population (**Figure 4B**). The Alum/α-GC combination caused a variable increase in the PD-1^hi^/CXCR5^hi^ population which was not statistically significant (**Figure 4B**). The 7DW8-5 adjuvant and the Alum/7DW8-5 combination did not significantly increase frequencies or total numbers of iNKT cells, PD-1^lo^/CXCR^lo^ or PD-1^hi^/CXCR5^hi^ iNKT cells. These data show that α-GC and the Alum/α-GC combination in the amounts given expand PD-1^+^/CXCR5^+^ iNKT cells but poorly stimulate expansion and differentiation of functional follicular helper PD-1^hi^/CXCR5^hi^ iNKT cells.

### Transient Toxicity on Combining Alum and CD1d-Binding Adjuvants

**Figure 5:**
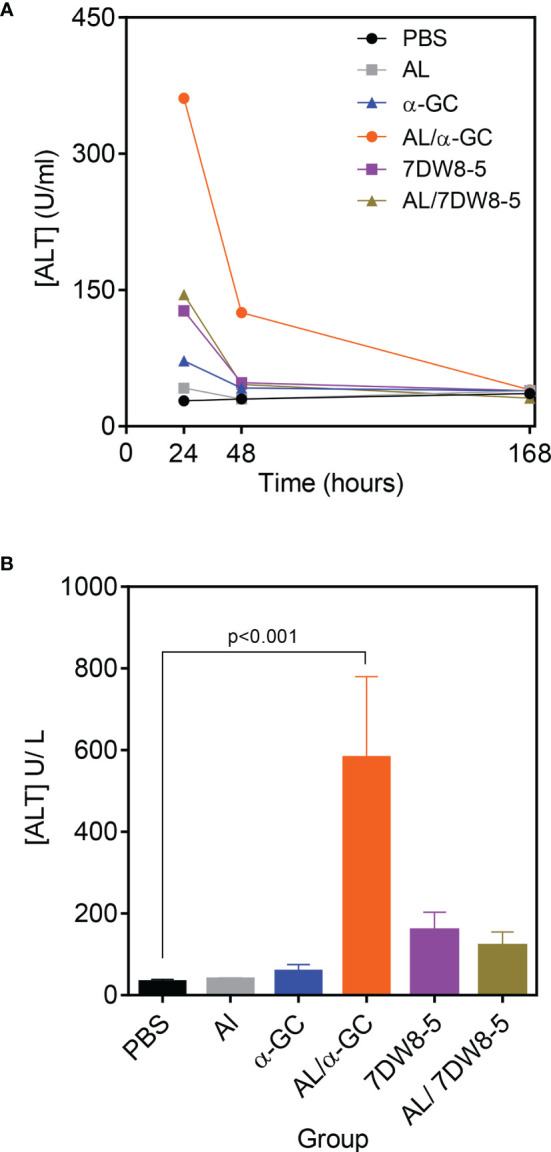
Transient hepatotoxicity on combining Alum and CD1d-binding adjuvants. B6 mice were immunized s.c. with adjuvants indicated singly or in combinat**i**on and in doses used for other experiments in the study. Heparinized blood samples were collected and pooled and analyzed as described in materials and methods. **(A)** Graph shows ALT concentration over time and is representative of four independent experiments. Each data point represents pooled blood samples from 3 individual mice. **(B)** Shows the mean ± SD ALT concentration for 4 experiments at the 24 hr time point. Data were analyzed by ANOVA followed by Dunnett’s post-test.

Following immunization with Alum, or α-GC, significant hepatic toxicity could not be detected in response to either agent and was evidenced by maintenance of baseline levels of Alanine Transferase (ALT) on days 1, 2 and 7 following immunization (**Figure 5A**). In contrast, 7DW8-5 alone, the combination of Alum/α-GC and of Alum/7DW8-5 led to a transient increase in serum ALT concentration. The ALT concentrations were elevated on days 1 and returned to baseline by day 2 or between day 2 and 7 (**Figure 5A**). A comparison of ALT concentrations at their peak on day 1, revealed that the Alum/α-GC combination caused more ALT release than 7DW8-5 alone or the Alum/7DW8-5 combination (**Figure 5B**).

Several other factors were not significantly affected by the Alum/α-GC or the Alum/7DW8-5 combinations (**Table 1**). 7DW8-5 alone and both adjuvant combinations caused a temporary drop in Alkaline Phosphatase concentrations for reasons that are unclear. All adjuvants singly and in combination caused a temporary drop in blood glucose concentration which only reached significance with the Alum/α-GC combination. 7DW8-5 alone and the Alum/7DW8-5 combination caused elevations in globulin concentration, but responses were variable and non-significant. This may indicate inflammation following adjuvant administration. These data show that the combination of Alum and CD1d-binding adjuvants resulted in transient hepatotoxicity, and which resolved quickly after immunization. Other factors were not altered to a significant degree, indicating that the adjuvant combination appeared to be generally well-tolerated.

**Table 1:** Blood analysis following administration of adjuvants singly and in combination.

	PBS			Alhydrogel			α-GC			Units
	Mean	SD	n	Mean	SD	n	Mean	SD	n	
Albumin	4.27	0.06	3	4.27	0.25	3	4.23	0.06	3	g/dL
Alkaline Phosphatase	105.33	6.51	3	94.67	15.82	3	101.33	7.57	3	U/L
Alanine Transferase	33.67	4.93	3	40.00	2.65	3	59.33	15.53	3	U/L
Amyloid P	623.33	38.70	3	686.67	57.62	3	634.00	56.93	3	U/L
Total Bilirubin	0.30	0.10	3	0.33	0.06	3	0.43	0.23	3	mg/dl
Blood Urea Nitrogen	21.33	3.21	3	22.67	3.51	3	22.67	5.03	3	mg/dl
Calcium	9.87	0.23	3	10.10	0.20	3	10.10	0.00	3	mg/dl
Phosphate	5.50	0.40	3	5.70	0.66	3	6.10	0.61	3	mg/dl
Creatinine	0.27	0.06	3	0.30	0.18	3	*<0.2*	na	3	mg/dl
Glucose	211.33	19.14	3	173.67	19.86	3	169.00	11.79	3	mg/dl
Na^+^	147.33	3.21	3	152.67	2.08	3	150.67	2.08	3	mmol/L
K^+^	6.10	0.00	3	6.17	0.38	3	5.93	0.15	3	mmol/L
Total Protein	5.23	0.12	3	5.37	0.15	3	5.33	0.15	3	g/dL
Globulin	0.93	0.06	3	1.13	0.12	3	1.07	0.25	3	g/dL
	AL/α-GC			7DW5-8			AL/7DW5-8			**Units**
	**Mean**	**SD**	**n**	**Mean**	**SD**	**n**	**Mean**	**SD**	**n**	
Albumin	3.83	0.058	3	3.70	0.42	2	3.60	0.85	2	g/dL
Alkaline Phosphatase	78.67 *	*12.01*	*3*	63.50 *	*6.36*	*2*	70.00 *	*14.14*	*2*	U/L
Alanine Transferase	582.67 ***	196.84	3	158.50	44.55	2	121.00	33.94	2	U/L
Amyloid P	597.33	58.60	3	597.00	1.41	2	828.00	417.19	2	U/L
Total Bilirubin	0.33	0.06	3	0.30	0.00	2	0.30	0.00	2	mg/dl
Blood Urea Nitrogen	18.33	5.86	3	23.50	0.71	2	20.00	1.41	2	mg/dl
Calcium	10.07	0.12	3	10.15	0.07	2	10.20	0.14	2	mg/dl
Phosphate	4.93	0.49	3	5.15	0.21	2	5.20	0.00	2	mg/dl
Creatinine	*<0.2*	na	3	0.30	0.14	2	0.25	0.07	2	mg/dl
Glucose	140.33 *	27.02	3	159.50	53.03	2	176.50	30.41	2	mg/dl
Na+	150.00	0.00	3	152.00	0.00	2	150.50	0.71	2	mmol/L
K+	5.67	0.15	3	6.15	0.07	2	6.15	0.49	2	mmol/L
Total Protein	5.17	0.058	3	5.50	0.14	2	5.40	0.14	2	g/dL
Globulin	1.33	0.058	3	1.75	0.49	2	1.85	0.78	2	g/dL

Data shows mean + SD values for the metabolites and proteins indicated. Replicates indicated (n) refer to the number of independent experiments but in each experiment blood samples from 3 mice were pooled to generate sufficient volume for analysis. Values for creatinine (italics) were below the limits of detection (<0.2 mg/dL) in the α-GC and Alum/ α-GC samples. Statistically significant decreases are shown in italic orange and increases are shown in bold purple as determined by one-was ANOVA with Dunnett’s post-test. (*p,0.05, ***P < 0.001).

### Humoral Immunity Using Alum and 7DW8-5 in a Prime Boost Strategy

Since additive or synergistic effects of administering Alum and α-GC or 7DW8-5 together were not observed, mice were subject to a prime-boost strategy in which they received NP-KLH/Alum in an initial vaccine then NP-KLH/7DW8-5 in a booster vaccine (**Figure S4**). Controls included omission of the adjuvant from the prime and from the booster vaccine. IgM and IgG2c titers were observed to be uniform across all experimental groups. Clear adjuvant effects of Alum in the priming dose were observed for IgG1, IgG2b, and IgG3. However, 7DW8-5 exerted no additional effect on titers when administered in the booster vaccine. Absence of adjuvant in the priming dose followed by inclusion of 7DW8-5 in the booster failed to exert a significant adjuvant effect on titers. Therefore, as expected, Alum exerted a strong adjuvant effect on Th2 responses (IgG1) and 7DW8-5 when included in a booster vaccine did not alter the IgG subclass profile.

Another group of mice was immunized with NP-KLH/7DW8-5 in the priming dose with NP-KLH/Alum being administered as the booster dose, reversing the order from the previous experiment. Comparison of these two groups revealed no difference in IgM or IgG3 titers in primary or secondary bleeds (**Figure S5**). In contrast, 7DW8-5 was less effective at stimulating production of IgG1, IgG2b, and IgG2c than Alum as evidenced by primary bleed titers. Upon completion of the immunization schedule, secondary bleed titers revealed that the order in which the adjuvants was administered had no effect on IgM, IgG1, or IgG3 production. However, administration of 7DW8-5 first led to lower IgG2b and IgG2c titers than administration of Alum first. This data therefore suggests that the order in which adjuvants are administered affected Ig class switch and thus the overall production of Th1-driven subclasses (IgG2c).

## Discussion

We have shown that the combination of Alum (Alhydrogel™) with α-GC or 7DW8-5 did not confer substantial advantage with regards to protection against *C. difficile*. This was the case using relatively low amounts of the CTD antigen and Alum which proved to be quite effective. Examination of Ab subclasses revealed that inclusion of α-GC led to increased production of IgG1, IgG2b, and IgG2c, perhaps altering the Th2/Th1 balance induced by Alum. Although this did not translate to advantages in protection against *C. difficile*, this broadening of the Ab subclasses could be useful in other infectious disease contexts where a Th1-driven Ab response can be beneficial.

Inclusion of α-GC or 7DW8-5 in an Alum/CTD vaccine conferred the same protection against *C. difficile* and was associated with similar IgG1, IgG2b, and IgG2c profiles. However, α-GC was more efficient at stimulating iNKT cell expansion than 7DW8-5. Interestingly, 7DW8-5 and Alum/7DW8-5 did not stimulate increases in PD-1^+^/CXCR5^+^ or PD-1^hi^/CXCR5^hi^ (iNKTfh) cells. In a previous report, we demonstrated that iNKT cell expansion following α-GC treatment was a product of proliferation and differentiation (46), which contrasts with 7DW8-5. The localized versus systemic distribution of 7DW8-5 and α-GC respectively (44) may be responsible for differential iNKTfh expansion. This is because different CD1d-expressing APCs could present the ligand to distinct iNKT populations in different environments and exert distinct effects on their expansion and differentiation into iNKT cells. This suggests that different CD1d ligands could be engineered to expand iNKTfh cells or to avoid this effect dependent on the response required.

There are several reports that the iNKT cell population can influence memory to protein antigens (17, 21, 27). Thus far it remains unclear whether iNKTfh cells are associated with enhanced memory B cell responses against protein antigens. Some information is available regarding iNKTfh cells and polysaccharide antigens. The Bendelac group demonstrated iNKT/iNKTfh cell-driven anti-polysaccharide responses (31). They reported that immunization with capsular pneumococcal polysaccharides and α-GC resulted in class-switch recombination, affinity maturation and B cell memory but with a limited expansion of iNKTfh cells (31). We were unable to observe convincing Ab recall responses to T-independent carbohydrate Ags co-administered with α-GC although increased iNKTfh expansion, primary Ab responses and class switch were observed (67). Taken together, these studies suggests that iNKTfh cells provide additional B cell help for anti-polysaccharide responses but do not induce B cell memory.

Administration of Alum, α-GC, and 7DW8-5 singly did not result in overt signs of toxicity as evidenced by stable concentrations of serum ALT and several other metabolites and serum proteins. In contrast, the Alum/α-GC combination resulted in a transient elevation of ALT. Approximately 30% of murine hepatic lymphocytes are iNKT cells (68). In humans, the frequency of iNKT cells in liver is much lower at around 0.5% (68) and α-GC has been well-tolerated in the clinic in experimental cancer immunotherapies (39). The Alum/α-GC combination may not be of concern in terms of future administration to humans, but our results do suggest that the systemic distribution of α-GC in combination with Alum can result in subsequent NKT-dependent damage to hepatocytes. Crucially, toxicity can be minimized using 7DW8-5 which remains more localized than α-GC (44).

The data herein, suggest that inclusion of α-GC or its analogs in Alum-based vaccines could be optimized and applied to vaccination against *C. difficile* or other pathogens. We recently reported that α-GC is a good adjuvant for stimulating protection against *C. difficile* when surface polysaccharide II (PSII), a T-independent antigen is used for immunization (63). In that study, anti-PSII IgG1 was observed using a PSII/α-GC vaccine and crucially lowered the bacterial burden. Arguably, an Alum/CTD prime followed by a PSII/α-GC booster could be a useful strategy, although as our initial data show, the order of events in a prime boost strategy may be important.

Our findings therefore suggest that α-GC could be optimized for inclusion in existing adjuvant platforms but toxicity, desired Ab subclass, and the order of events in a prime boost strategy will need to be examined. The physicochemical properties of Alum formulations including Alhydrogel such as size, shape, charge, hydration, antigen adsorption, and aggregation can all be manipulated [reviewed in (69)]. Arguably, the interaction of Alum with glycolipid adjuvants warrants investigation and optimization.

## Data Availability Statement

The raw data supporting the conclusions of this article will be made available by the authors, without undue reservation.

## Ethics Statement

The animal study was reviewed and approved by OUHSC Institutional Animal Care and Use Committee.

## Author Contributions

GL designed and performed experiments, analyzed data, and wrote the manuscript. KB-N performed experiments, analyzed data, and edited the manuscript. SA performed experiments. TS performed experiments. JB provided critical resources and input on experiment method and edited the manuscript. ML conceived and designed the project, designed and assisted with experiments, analyzed data, and wrote the manuscript. All authors contributed to the article and approved the submitted version.

## Funding

This work was supported by NIH grants AI134719 (ML) and AI119048 (JB).

## Conflict of Interest

The authors declare that the research was conducted in the absence of any commercial or financial relationships that could be construed as a potential conflict of interest.

## Publisher’s Note

All claims expressed in this article are solely those of the authors and do not necessarily represent those of their affiliated organizations, or those of the publisher, the editors and the reviewers. Any product that may be evaluated in this article, or claim that may be made by its manufacturer, is not guaranteed or endorsed by the publisher.
