# Supplementary material for: Use of a Clostridioides difficile Murine Immunization and Challenge Model to Evaluate Single and Combination Vaccine Adjuvants Consisting of Alum and NKT Cell-Activating Ligands
Source: Front Immunol. 2022 Jan 14;12:818734. doi: 10.3389/fimmu.2021.818734 (PMC8794951; doi:10.3389/fimmu.2021.818734)
Supplement: Supplementary file 1 [file DataSheet_1.pdf]

SUPPLEMENTAL FIGURE LEGENDS

Fig. S1 Vaccination Schematics and Timelines (A) Outlines the workflow for the experiments described in Figs. 1 and 2. (B) Provides a similar outline for the experiments in Figs. S3 and S4.

Fig. S2 Detection of iNKT cells and dNKT cells by flow cytometry. (A) Distinguishes specific and non-specific binding of detection of PBS57-loaded and empty CD1d tetramer to splenocytes. The iNKT population is readily detected using the PBS57-loaded tetramer. (B) Shows workflow for detection total NKT cells (iNKT and dNKT, $TCR\beta^+$, $NK1.1^+$) and iNKT cells ($TCR\beta^+$, $NK1.1^+$, $tetramer^+$). (C) Shows that iNKT and dNKT cells are detected within live cell singlet lymphocyte populations. Numbers in purple depict sequential steps in gating process.

Fig. S3 NKT cell frequencies. As an accompaniment to Figure 3, (A) depicts frequencies of splenic iNKT (upper panel) and dNKT cells (lower panel). As an accompaniment to Figure 4, (B) depicts frequencies of lymph node iNKT cells (upper panel), $PD1^+/CXCR5^+$ iNKT cells (middle panel) and $PD1^{hi}/CXCR5^+$ iNKT cells (lower panel). One way ANOVA with Dunnett's post-test was used to detect significant differences (*<0.05).

Fig. S4 Using Alum and 7DW8-5 in a prime boost strategy did not enhance humoral immunity. Mice were administered prime and boost vaccines as indicated before collection of blood and preparation of plasma samples. Antigen-specific IgM, IgG1, IgG2b, IgG2c, and IgG3 were then detected by ELISA. Graphs depict endpoint titers showing geometric means and each data point represents an individual mouse. Significant differences were detected by one-way ANOVA and Dunn's post-test (*, P<0.05, **, P<0.01).

Fig. S5 Use of 7DW8-5 adjuvant in a priming vaccine alters Ig subclass profile Samples from Group D of Fig. 2 (NP-KLH/Alum prime, NP-KLH/7DW8-5 boost) were re-run in the ELISA with Group E (NP-KLH/7DW8-5 prime, NP-KLH/Alum boost). Primary bleed sera (post-prime, pre-boost) and secondary bleed sera (post-boost) were analyzed. Graphs depict endpoint IgM, IgG1, IgG2b, IgG2c, and IgG3 titers with geometric means. Each data point represents an individual mouse. Significant differences were detected by Mann Whitney U test.

(A)

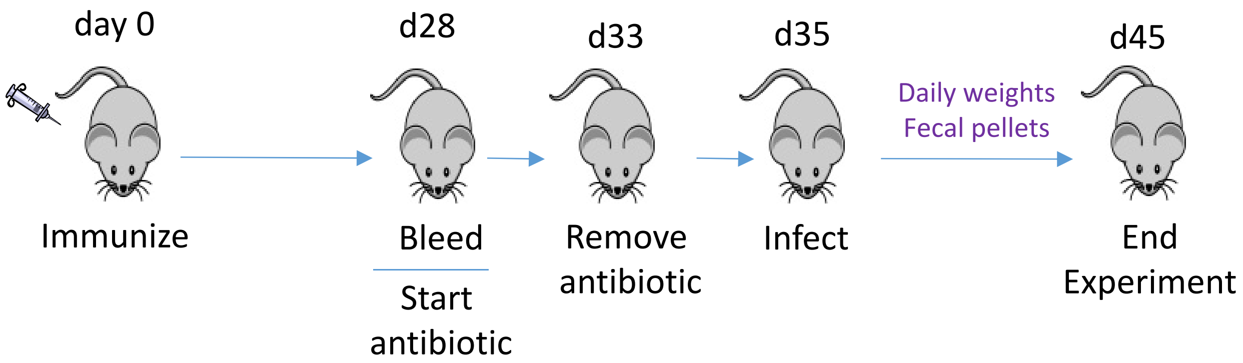
day 0
d28
d33
d35
d45
Daily weights
Fecal pellets
Immunize
Bleed
Start antibiotic
Remove antibiotic
Infect
End Experiment

(B)

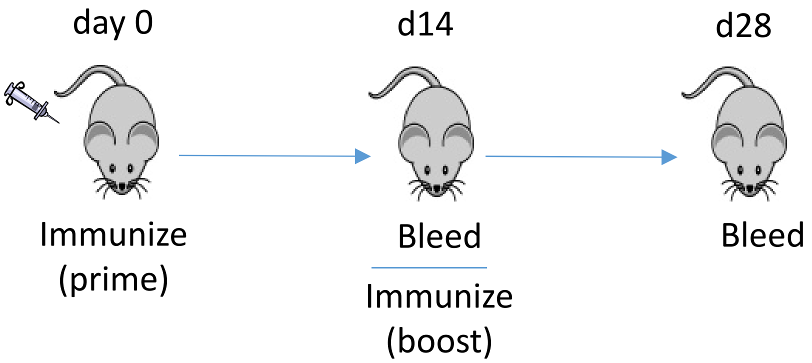
day 0
d14
d28
Immunize (prime)
Bleed
Immunize (boost)
Bleed

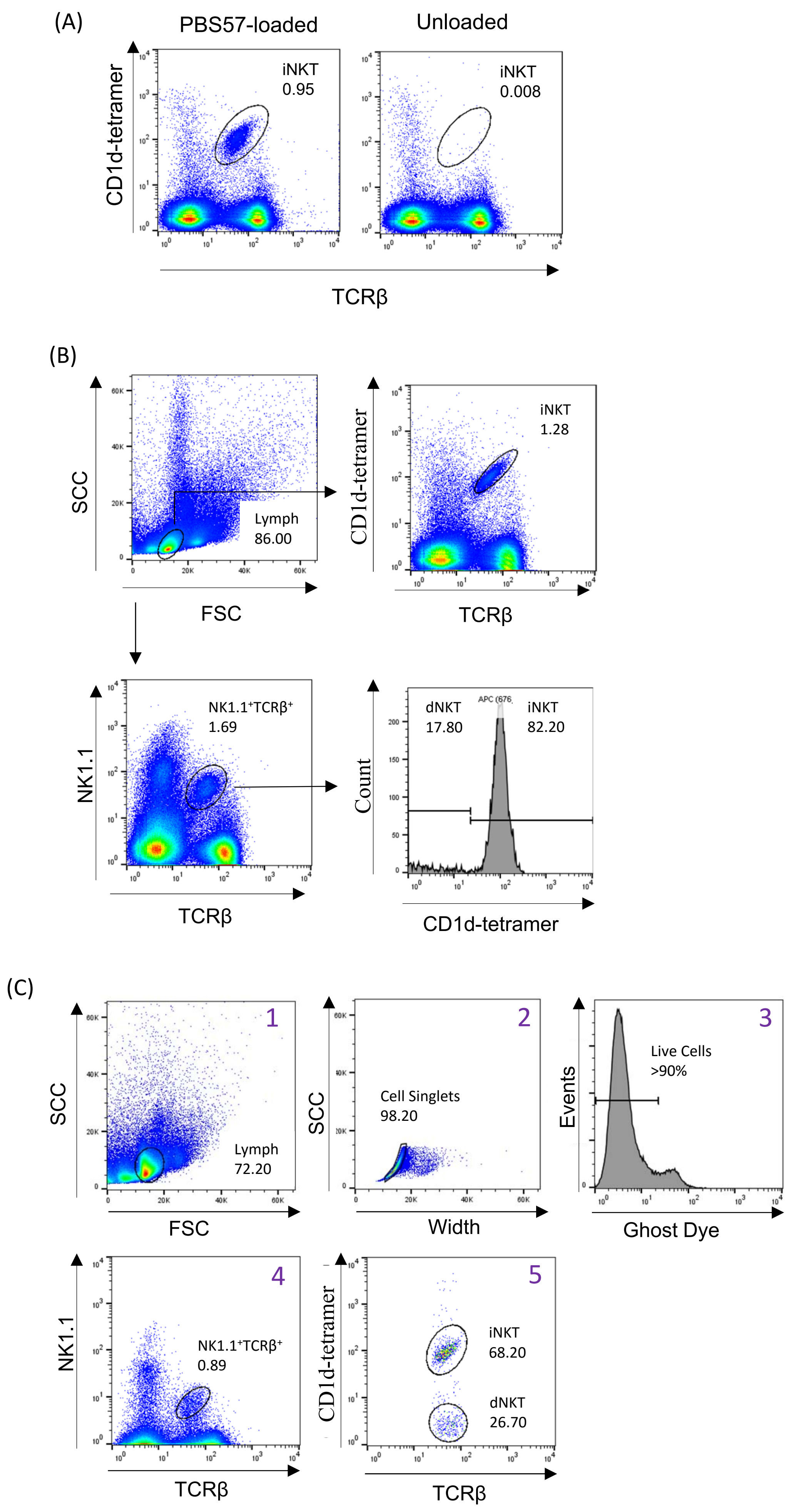
(A)
PBS57-loaded
Unloaded
CD1d-tetramer
iNKT
0.95
iNKT
0.008
TCRβ
(B)
SCC
FSC
Lymph
86.00
CD1d-tetramer
iNKT
1.28
TCRβ
NK1.1
NK1.1⁺TCRβ⁺
1.69
TCRβ
Count
dNKT
17.80
iNKT
82.20
CD1d-tetramer
(C)
1
SCC
Lymph
72.20
FSC
2
SCC
Cell Singlets
98.20
Width
3
Events
Live Cells
>90%
Ghost Dye
4
NK1.1
NK1.1⁺TCRβ⁺
0.89
TCRβ
5
CD1d-tetramer
iNKT
68.20
dNKT
26.70
TCRβ

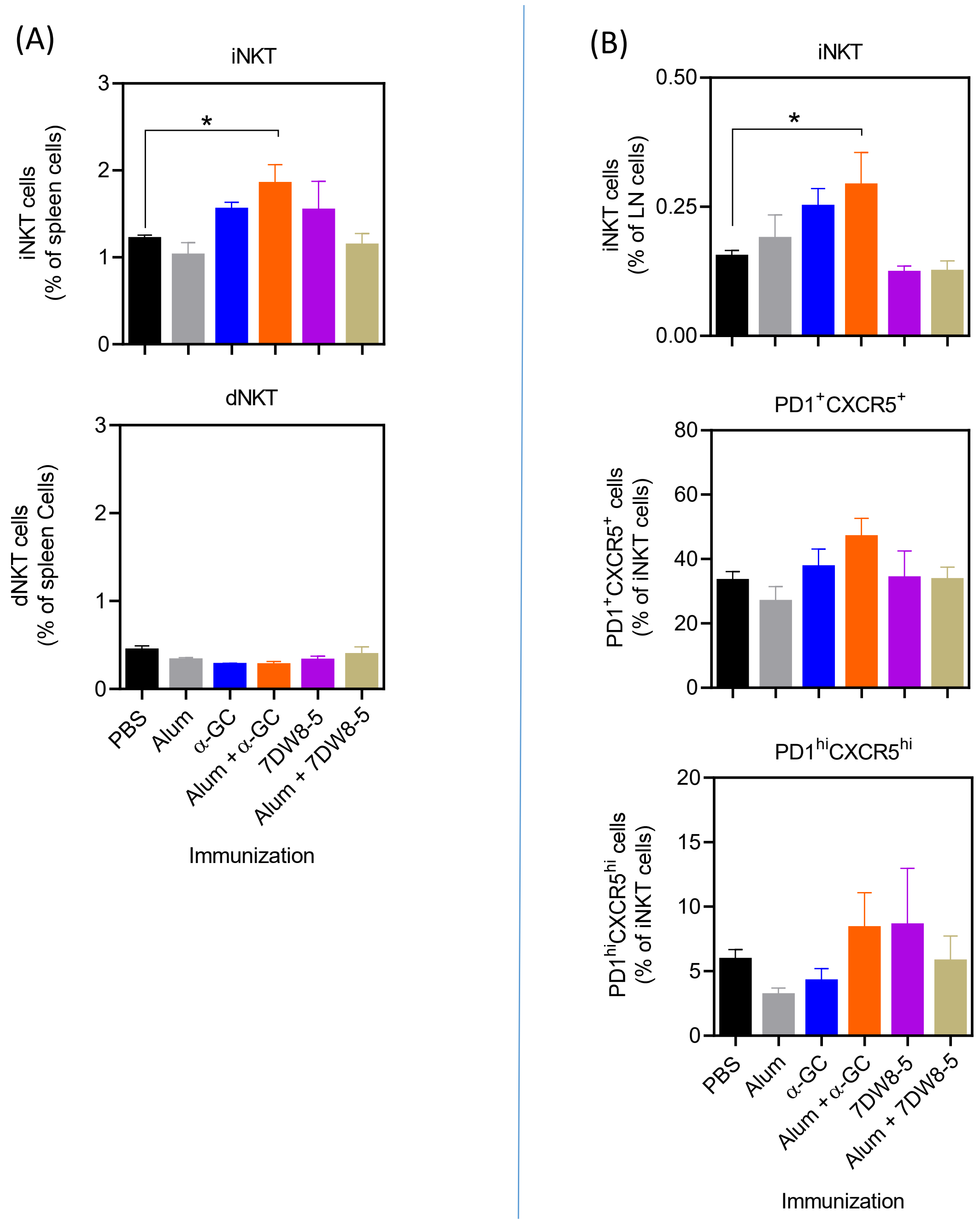
(A)
iNKT
iNKT cells (% of spleen cells)
*
dNKT
dNKT cells (% of spleen Cells)
PBS
Alum
α-GC
Alum + α-GC
7DW8-5
Alum + 7DW8-5
Immunization
(B)
iNKT
iNKT cells (% of LN cells)
*
PD1+CXCR5+
PD1+CXCR5+ cells (% of iNKT cells)
PD1hiCXCR5hi
PD1hiCXCR5hi cells (% of iNKT cells)
Immunization

Lang et al Figure S4

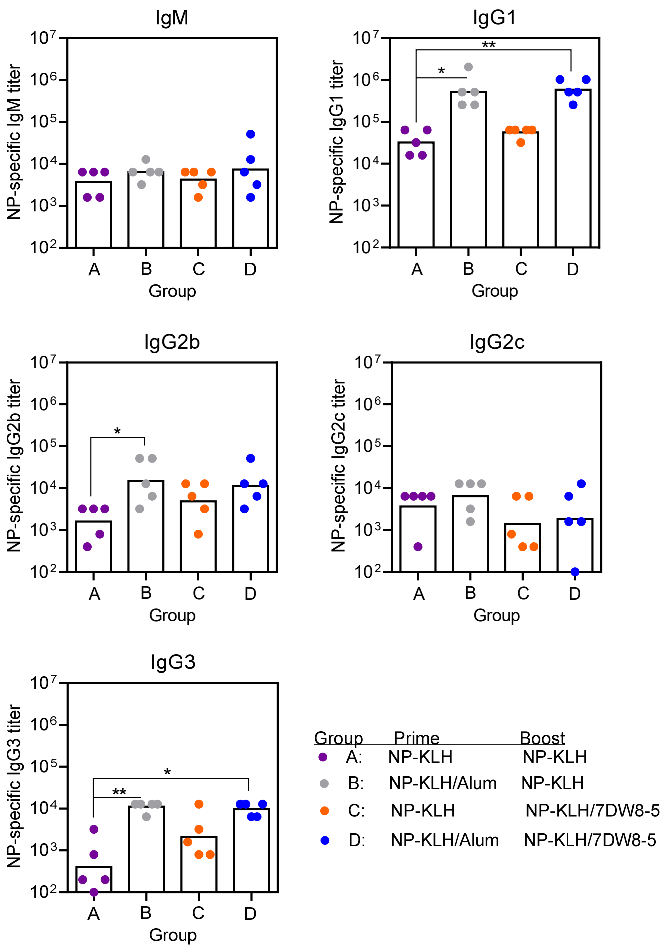

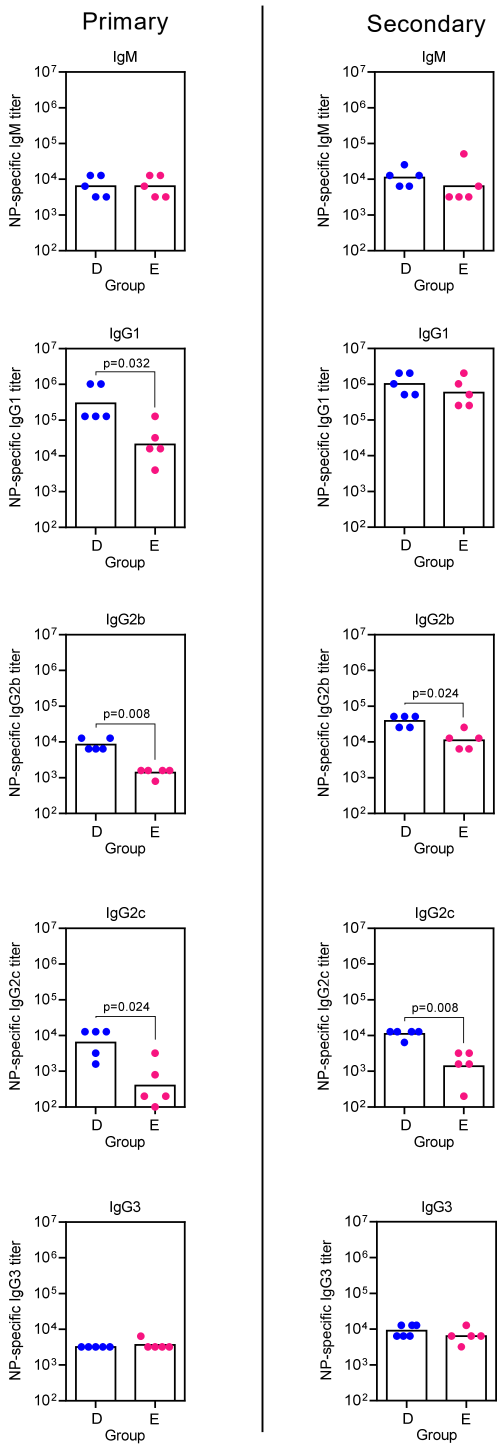

Group	Prime	Boost
D:	NP-KLH/Alum	NP-KLH/7DW8-5
E:	NP-KLH/7DW8-5	NP-KLH/Alum
